# Effects of scalable, wordless, short, animated storytelling videos on hope in China: a nationwide, single-blind, parallel-group, randomised controlled trial

**DOI:** 10.7189/jogh.15.04140

**Published:** 2025-06-20

**Authors:** Wenjin Chen, Maya Adam, Pascal Geldsetzer, Lirui Jiao, Jennifer Gates, Jinghan Zhao, Till Bärnighausen, Simiao Chen, Chen Wang

**Affiliations:** 1 School of Population Medicine and Public Health, Chinese Academy of Medical Sciences & Peking Union Medical College, Beijing, China; 2 Heidelberg Institute of Global Health (HIGH), Faculty of Medicine and University Hospital, Heidelberg University, Heidelberg, Germany; 3 Department of Pediatrics and Center for Digital Health, School of Medicine, Stanford University, Stanford, California, USA; 4 Department of Medicine, Stanford University, Stanford, California, USA; 5 Department of Global Health and Population, Harvard T.H. Chan School of Public Health, Boston, Massachusetts, USA; 6 Department of Health Policy and Management, Gillings School of Global Public Health, University of North Carolina at Chapel Hill, Chapel Hill, North Carolina, USA; 7 Icahn School of Medicine, Mount Sinai, New York, New York, USA

## Abstract

**Background:**

People with higher levels of hope are more likely to be vaccinated. Short, animated story (SAS) videos have shown promise for communicating health messages and boosting hope in certain populations. We explored the potential of scalable SAS vaccine promotion videos for boosting hope levels among Chinese adults.

**Methods:**

In this single-blind, parallel-group, randomised controlled trial, we recruited adults from China through quota sampling. Participants were randomly assigned in a 1:1:1:1 ratio to one of three SAS video intervention groups (humour, analogy, or emotion) or a control group. After watching the videos or being assigned to the control group, participants completed the Adult Hope Scale. Level of hope among participants was compared between each intervention group and the control group, as well as among the different intervention groups, with *P*-values adjusted for multiple comparisons.

**Results:**

We included 12 000 participants aged 18 and above, residing in China, in our analysis. In the main analysis and sensitivity analyses, no significant intervention effects were observed in any of the three intervention groups. Furthermore, comparisons among the intervention groups showed no significant differences, indicating no variation in the effects of the three intervention videos. In subgroup analyses, however, we observed significant differences among regional subgroups (*P* < 0.05), with Video A (humour) boosting hope in participants from the southern and southwestern regions, when compared with other regions.

**Conclusions:**

While the short, single-exposure SAS videos did not significantly enhance overall hope levels among Chinese adults, the effectiveness of humour in certain subgroups highlights the cultural adaptability of health communication strategies. Given its scalability and accessibility, this approach warrants further research to refine narrative techniques, optimise engagement across diverse populations, and explore its broader application in global health communication.

Hope is the perceived capacity to build pathways toward our goals and motivate ourselves to use those pathways [1]. Like other psychological constructs such as optimism, self-efficacy, and self-esteem, hope is a measurable parameter that has been empirically linked to various positive outcomes, including enhanced health, psychosocial well-being, and academic performance [1]. Recent research also suggests that hope may be a critical predictor of vaccine uptake. Studies conducted in various geographic regions, including China, have found that individuals with higher levels of hope are more likely to be vaccinated against COVID-19 [2–5]. These findings raise an interesting question: could interventions aimed at boosting hope augment public health strategies aimed at reducing vaccine hesitancy?

For decades, researchers have studied various approaches to addressing vaccine hesitancy, yet lack of confidence in vaccines remains a major challenge, and effective strategies to improve confidence continue to elude the public health community [6,7]. During the COVID-19 pandemic, surges of hesitancy impeded efforts to contain the disease in many parts of the world and underscored the wide variety of complex and context-specific factors fuelling vaccine hesitancy [8]. Amidst the global scramble to identify effective methods of boosting COVID-19 vaccine uptake, increasing hope emerged as a potentially important target for public health communication strategies [9].

Entertainment media, including films and short videos depicting underdogs or characters who struggle to achieve goals despite challenging circumstances, have proven effective for boosting hope and motivation [10]. Researchers have explored whether entertainment media that is grounded in hope theory and related theoretical foundations might be used to offset the negative effects of stress on well-being [11]. Moreover, research on short-form ‘edutainment’ (short, animated storytelling videos) in a large sample of German adults has shown that even small doses (*i.e*. videos under six minutes in length) can significantly nurture hope [12].

In China, as in many countries, vaccine hesitancy has undermined the efficacy of nationwide vaccination programmes to contain COVID-19, resulting in a substantial avoidable toll on the economy and society. Addressing vaccine hesitancy in China is therefore an ongoing public health priority [13]. In China, specific cultural barriers significantly impact vaccine acceptance, including the spread of misinformation during the pandemic, population heterogeneity, collectivist cultural values, and the digital divide in accessing health information. First, social media and other informal channels facilitated the spread of misinformation during the pandemic, undermining public trust in vaccines and exacerbating vaccine hesitancy [14]. Second, due to China's vast population and strong demographic heterogeneity, the effectiveness of health communication campaigns may vary significantly between urban and rural areas [15]. Additionally, as a predominantly collectivist society, China places a strong emphasis on communal well-being over individual interests, making health messages framed around collective responsibility more persuasive [16]. However, this advantage may be offset by the digital divide, further exacerbating inequalities in health information access. For instance, older adults, due to lower digital literacy, limited internet access, and unfamiliarity with smart devices, face significant challenges in obtaining health information, which affects the fairness and effectiveness of vaccine decision-making [17]. Given these cultural and social factors, designing health communication strategies that are both rapid and scalable is particularly critical.

Short, animated storytelling (SAS) is an emerging entertainment-education approach has shown promise for addressing vaccine hesitancy in some populations [18–21]. Such an approach is operationalised in SAS videos, which utilise a blend of visual storytelling, compelling sound design, and educational content to deliver health information in an engaging and relatable manner [19,22,23]. When developing SAS videos, best practices from psychology, public health, media studies, and communication are often integrated to ensure that the content is both scientifically accurate and engaging. Because SAS videos can be designed to be wordless and take a primarily visual approach to storytelling, they are able to transcend language and cultural barriers, enhancing their accessibility [24]. Since these videos are cost-effective to produce and easy to scale rapidly across large, diverse audiences, they are well-suited for countries, such as China, with many culturally and linguistically diverse sub-populations [25]. However, their mechanism of action – including their hypothesised capacity to boost hope in diverse populations – remains understudied. Since SAS videos have shown promise for both addressing vaccine hesitancy in the Chinese context, and boosting hope in other global settings [12], this study explored the effect of SAS videos on hope in the Chinese context. We hypothesised that a single exposure to an SAS video about vaccine hesitancy could measurably boost hope in a diverse sample of Chinese participants.

The main objective of our study was to assess the effect of scalable SAS videos on hope scores among Chinese adults, using a nationwide, single-blind, parallel-group, randomised controlled trial. The findings of this study could support the ongoing distribution of SAS video interventions and guide the optimisation of future entertainment-education interventions to increase hope and boost vaccination rates in the general public.

## METHODS

### Study design

We implemented a nationwide, single-blind, parallel-group, randomised controlled trial. Interventions were delivered and data were collected via computer web pages or smartphones. Participants in the intervention group submitted their sociodemographic information before being randomly assigned to watch one of three animated videos. After intervention-group participants watched their videos, their hope scores were measured using a validated survey customised for our participant population. By contrast, control group participants took the survey first and were then provided with access to the video interventions post-trial. The study obtained ethical approval from the Ethics Committee of the Basic Medical College at Peking Union Medical College on 10 March 2021 (identifier: 062-2021). The trial was listed in the German Clinical Trials Register on 9 February 2021 (identifier: DRKS #00023650).

### Participants

Our goal was to recruit 12 000 Chinese adults aged 18 years and above, using quota sampling to ensure national representation in terms of age, gender, and residence in urban or rural areas (Text S1 in the **Online Supplementary Document**) [26–28]. The quotas were derived from the 2019 population estimates by the National Bureau of Statistics of China [29]. Participants were enlisted through KuRunData (https://www.kurundata.com), a market research firm with over 17 million registered members in China. Sampling was conducted from this extensive pool. Recruitment was executed via multiple channels to ensure the diversity and representativeness of the data. These channels included KuRunData's proprietary software, digital network advertisements, internet searches, word of mouth, membership referrals, social media platforms such as TikTok, partner recommendations, and over 200 national coordinators who facilitated offline recruitment. These varied sources significantly reduced reliance on any single demographic group.

Invited respondents were not informed of the survey topic before they provisionally expressed interest in participating. After expressing interest, the study's purpose and procedures were introduced on the informed consent page. Participants were then provided with ethically approved information about the study and asked to confirm consent online.

### Randomisation and masking

Participants who enrolled in the study were randomly assigned to three video intervention groups and one control group in a 1:1:1:1 ratio using a computer-generated sequence that was independent of the researchers. Each intervention group participant watched one of the three designated videos, without knowledge of the other groups. Every participant received a unique, anonymous ID and completed a set of validated surveys to collect data. KuRunData handled data collection, ensuring participant anonymity to the research team, which accessed only deidentified data during analysis. The research team remained blinded to the individual participant assignments throughout the trial and data analysis phases.

### Enrolment procedures

The study recruitment took place from 15 April–25 May 2021. Potential participants were required to register an account on KuRunData and provide personal information. After agreeing to participate, they first answered sociodemographic questions, including on their age, gender, residence (urban or rural), education level, household annual income, and province. Then, intervention group participants watched their assigned animated video and completed the hope scale measure (outcome measure) on either a computer or a smartphone. Control group participants completed the hope survey first and were then offered voluntary, post-trial access to the intervention videos. All steps were completed in a single session. Participants received a reward of five Chinese Yuan (CNY) upon completing the survey.

### Intervention video development

**Figure 1:**
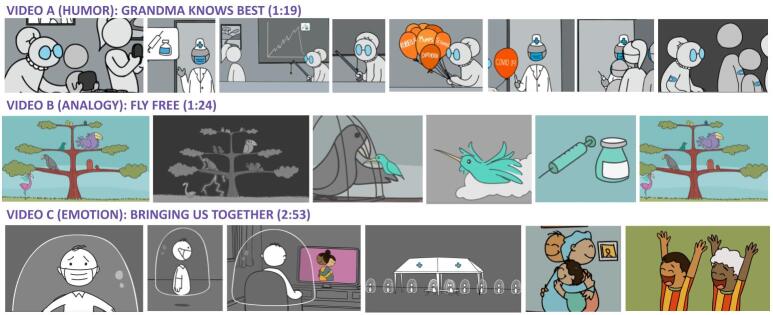
Selected screenshots from the three intervention videos.

We tested three short, animated entertainment-education videos between one and three minutes in length. Selected screenshots from the video interventions are featured in **Figure 1**. Each SAS video features a different instructional storytelling approach. Video A, ‘Grandma Knows Best’, employs humour to engage viewers in the story of a comically determined grandmother who tries to educate her family and convince them to accept COVID-19 vaccination. Video B, ‘Fly Free’, employs analogy as an instructional approach, with caged birds representing people who were forced to stay home during the global COVID-19 lockdowns. In this video, the bird characters are freed once they decide to get vaccinated. Video C, ‘Bringing us Together’, employs an emotion-driven storyline featuring the story of a socially isolated main character, whose experiences aim to reflect widespread, global struggles with loneliness and isolation during the pandemic lockdowns. The character re-enters his community, after being vaccinated, and this is portrayed amidst a backdrop of widespread euphoria as lockdowns are lifted and freedom is restored.

The intervention videos were produced during the roll-out of the first COVID-19 vaccines, between November 2020–March 2021. We worked with an interdisciplinary team of experts – including professionals with expertise in the behavioural sciences, entertainment, and marketing – to integrate evidence-based vaccine-promoting messages into engaging and scalable short, animated entertainment-education videos. Character designs were culturally accessible and informed by our prior study, which involved respondents in 73 countries [30]. We applied principles of Universal Design for Learning to maximise inclusiveness, with slightly different approaches to inclusiveness applied in each of the three videos [31]. Video A relies on the use of iconic character representations that were free of cultural identifiers. We use different shades of grey to suggest variations in racial identity. Video B depicts diversity through the representation of various bird species. Video C uses a hybrid approach, with the main character depicted as an icon without cultural identifiers and the supporting characters depicted to represent diverse races/ethnicities.

Our team of creative partners reflected a global outlook. We also gathered input from various stakeholders in China, Mexico, Canada, Germany, USA, South Africa, and Australia. Additionally, throughout the development, feedback was solicited from a group of international vaccine-promotion experts. This feedback was shared, via WhatsApp and Zoom, with our creative partners, allowing them to revise their video planning documents and drafts. This process of responsive iteration (soliciting feedback from multiple stakeholders, and adapting the intervention in response to that feedback), is suggested in the human-centred design literature [32].

All the SAS intervention videos emphasised the efficacy of vaccines but did so through different storytelling approaches. Video A (humour) focused on the quirky grandmother character in an instructional-humour approach [33]. Video B (analogy) used a collection of colourful birds in a storytelling-analogy approach [34]. Video C (emotion) aimed to elicit emotion as a means of engagement [35]. The same 2D animation style was implemented by a single animator for all three intervention videos. None of the videos contained dialogue, so as to enhance their scalability across different languages and cultures. We chose this approach based on the observed rapid spread of earlier wordless, animated entertainment-education content [19]. Visual storytelling was supported by a compelling soundtrack in each video intervention. Through Universal Design for Learning principles, human-centred design, iterative optimisation, and dialogue-free storytelling, these design choices ensured the cultural neutrality of the animated videos, making them adaptable across diverse cultural contexts. The intervention videos can be viewed on YouTube at the following links:

1. Video A (humour): https://youtu.be/ap8xpyREaTc

2. Video B (analogy): https://youtu.be/fYYBJ0d6gl0

3. Video C (emotion): https://youtu.be/WH5KUhGtfa8

### Outcome

We used the Adult Hope Scale, which has been validated in the Chinese adult population, to assess participants' level of hope [36,37]. The scale includes 12 items, eight of which are scored items used to calculate the final hope score, and four are distractor items designed to maintain engagement and reduce response bias. Each item is rated 1–8, resulting in a total score ranging from 8–64. Higher scores indicate a higher level of hope. Additionally, participants scoring 46 or above are classified as the high hope group [38].

### Statistical analysis

Baseline characteristics included age, gender, residence, education level, household annual income, region, and economic belt, which were summarised as means (standard deviations, SDs) or n/n (%), with baseline comparisons performed using Pearson χ^2^ test for categorical variables and the Kruskal-Wallis test for continuous data. To reflect differences in socioeconomic development conditions across regions of China, we divided the country into four economic belts: East, Central, West, and Northeast [39]. As our main analysis, we first conducted a pooled comparison of participants in the three intervention groups against the control group, using independent *t* tests with two-tailed *P*-values and adhering to the intention-to-treat principle. Furthermore, we assessed hope scores through six comparisons (each intervention group *vs*. the control group and comparisons between intervention groups). We adjusted for multiple pairwise differences using the Bonferroni correction [40]. Statistical significance was considered at the 0.05-level, and the total alpha of 0.05 was evenly split across all co-primary comparisons, so only *P*-values (p_heterogeneity_) less than 0.0083 were deemed significant [41]. For each comparison, we calculated the absolute difference in means and Cohen's effect size d (Cohen's d) along with nominal 99.17% confidence intervals (CIs) to maintain an overall alpha level of 0.05. Statistical hypothesis testing was primarily conducted using parametric methods (*t* tests, χ^2^ tests) or non-parametric methods (Kruskal-Wallis rank sum test).

In the main analysis, we treated hope scores as continuous variables to evaluate the intervention's effect across the full range of hope levels. For the sensitivity analysis, we applied a binary classification by categorising participants with hope scores of 46 or higher as the 'high hope' group. This allowed us to investigate whether the intervention's effects observed in the continuous variable analysis remained consistent when using a threshold-based approach. By comparing the results of both methods, we aimed to ensure that the intervention's impact was robust and not dependent on the specific way hope was measured. The same significance level of 0.0083 was used for this comparison as in the main analysis.

We performed subgroup analyses across subgroups, with interaction *P*-values calculated using likelihood ratio tests. Interaction *P*-values (p_interaction_) below 0.05 were considered statistically significant. We distinguished interaction effects, which assess differences in intervention effectiveness between subgroups, from heterogeneity, which reflects variability in effectiveness within subgroups [42]. All statistical analyses were performed using *R*, version 4.1.2 (R Foundation for Statistical Computing, Vienna, Austria).

## RESULTS

### Sample characteristics

A total of 18 422 individuals expressed their interest in participating in the study. Of these, 2279 (12.37%) were excluded for not submitting questionnaires, 939 (5.10%) were excluded for being under 18 years old or not residing in China, and 2555 (13.87%) were excluded due to reaching full quotas. Ultimately, 12 649 (68.66%) participants were deemed eligible for the trial and were randomly assigned. Of these, 649 were excluded due to inadequate response times or their random selection as reserves to replace low-quality data. As a result, 12 000 participants (65.14%) were included in the final analysis (Figure S1 in the **Online Supplementary Document**). The participants were distributed across 31 of the 32 provinces, autonomous regions, and municipalities in mainland China (Figure S2 in the **Online Supplementary Document****).** The average age was 44.3 years (SD = 14.3), with 5873 (48.9%) women and 6127 (51.1%) men. Among all participants, 4744 (39.5%) resided in rural areas, 3600 (30.0%) had an education level of junior high or lower, 4411 (36.8%) had a family annual income under 90 000 Chinese Yuan, 3680 (30.67%) were in the Western region, and 4440 (37.0%) were in the West economic belt. Baseline characteristics were well balanced across the randomly assigned groups (**Table 1**). Furthermore, data from the study period indicated that only five participants (0.04%) had a history of COVID-19.

**Table 1:** Baseline characteristics across randomisation groups in total study population

Characteristics		Overall (n = 12 000)	Control group (n = 3000)	Video A (n = 3000)	Video B (n = 3000)	Video C (n = 3000)	*P*-value	
**Age, years (mean ± SD)**	44.3 ± 14.3		44.2 ± 14.4	44.2 ± 14.4	44.1 ± 14.1	44.7 ± 14.4	0.36
**Age group, n (%)**							0.73
18–29 y	2274 (19.0%)		585 (19.5%)	578 (19.3%)	558 (18.6%)	553 (18.4%)	
30–39 y	2355 (19.6%)		575 (19.2%)	578 (19.3%)	621 (20.7%)	581 (19.4%)	
40–49 y	2369 (19.7%)		593 (19.8%)	605 (20.2%)	589 (19.6%)	582 (19.4%)	
50–59 y	2290 (19.1%)		568 (18.9%)	561 (18.7%)	590 (19.7%)	571 (19.0%)	
≥60 y	2712 (22.6%)		679 (22.6%)	678 (22.6%)	642 (21.4%)	713 (23.8%)	
**Gender, n (%)**							0.25
Male	6127 (51.1%)		1504 (50.1%)	1521 (50.7%)	1578 (52.6%)	1524 (50.8%)	
Female	5873 (48.9%)		1496 (49.9%)	1479 (49.3%)	1422 (47.4%)	1476 (49.2%)	
**Residence, n (%)**							0.34
Rural	4744 (39.5%)		1163 (38.8%)	1211 (40.4%)	1210 (40.3%)	1160 (38.7%)	
Urban	7256 (60.5%)		1837 (61.2%)	1789 (59.6%)	1790 (59.7%)	1840 (61.3%)	
**Education, n (%)**							0.25
Junior high school or below	3600 (30.0%)		866 (28.9%)	903 (30.1%)	891 (29.7%)	940 (31.3%)	
High school or technical secondary school	3848 (32.1%)		994 (33.1%)	965 (32.2%)	931 (31.0%)	958 (31.9%)	
College diploma or above	4552 (37.9%)		1140 (38.0%)	1132 (37.7%)	1178 (39.3%)	1102 (36.7%)	
**Total household income, n (%)**							0.83
Below 90 000 CNY	4411 (36.8%)		1102 (36.7%)	1118 (37.3%)	1096 (36.5%)	1095 (36.5%)	
90 000 to 180 000 CNY	5172 (43.1%)		1296 (43.2%)	1306 (43.5%)	1278 (42.6%)	1292 (43.1%)	
Above 180 000 CNY	2417 (20.1%)		602 (20.1%)	576 (19.2%)	626 (20.9%)	613 (20.4%)	
**Region, n (%)**							0.71
Northeast China	1120 (9.3%)		272 (9.1%)	312 (10.4%)	270 (9.0%)	266 (8.9%)	
East China	2840 (23.7%)		717 (23.9%)	679 (22.6%)	737 (24.6%)	707 (23.6%)	
North China	2000 (16.7%)		504 (16.8%)	507 (16.9%)	478 (15.9%)	511 (17.0%)	
Central China	1200 (10.0%)		313 (10.4%)	299 (10.0%)	299 (10.0%)	289 (9.6%)	
South China	1160 (9.7%)		282 (9.4%)	309 (10.3%)	280 (9.3%)	289 (9.6%)	
Southwest China	1880 (15.7%)		453 (15.1%)	463 (15.4%)	488 (16.3%)	476 (15.9%)	
Northwest China	1800 (15.0%)		459 (15.3%)	431 (14.4%)	448 (14.9%)	462 (15.4%)	
**Economic belt, n (%)**							0.73
East China	4080 (34.0%)		1024 (34.1%)	1022 (34.1%)	1019 (34.0%)	1015 (33.8%)	
Central China	2360 (19.7%)		586 (19.5%)	583 (19.4%)	595 (19.8%)	596 (19.9%)	
Northeast China	1120 (9.3%)		272 (9.1%)	312 (10.4%)	270 (9.0%)	266 (8.9%)	
West China	4440 (37.0%)		1118 (37.3%)	1083 (36.1%)	1116 (37.2%)	1123 (37.4%)	

CNY – Chinese Yuan, SD – standard deviation

### Overall effectiveness assessment

**Figure 2:**
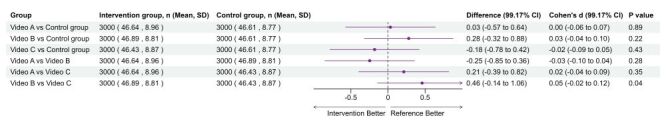
Effects of animated videos on hope. We made six comparisons, adjusting for multiplicity with an α-level of 0.0083 for each comparison; therefore, 99.17% CIs are reported to maintain an overall α-level of 0.05, and *P*-values below 0.0083 were considered statistically significant. CI – confidence interval.

In the main analysis, participants in the combined intervention groups did not show higher hope scores compared to those in the control group (mean difference = 0.04; 95% CI = −0.32, 0.41, *P* = 0.81). Specifically, Video A (humour; mean difference = 0.03; 99.17% CI = −0.57, 0.64, *P* = 0.89), Video B (analogy; mean difference = 0.28; 99.17% CI = −0.32, 0.88, *P* = 0.22), and Video C (emotion; mean difference = −0.18; 99.17% CI = −0.78, 0.42, *P* = 0.43) showed no significant effects on hope scores, indicating no improvement in any intervention group compared to the control group (**Figure 2**). In the sensitivity analysis examining high hope levels, no significant differences were found between the intervention and control groups (Figure S3 in the **Online Supplementary Document**). Additionally, the comparisons between the intervention groups were also not significant, indicating no notable differences in the effects of the different intervention videos (**Figure 2**; Figure S3 in the **Online Supplementary Document**).

### Subgroup analysis

We conducted subgroup analyses for each comparison: Video A (humour) *vs*. control group, Video B (analogy) *vs*. control group, and Video C (emotion) *vs*. control group. Forest plots illustrating the effectiveness of each animated video compared to the control group, stratified by age, gender, residence, education level, household annual income, region, and economic belt, are presented in Figure S4–6 and Figure S7–9 in the **Online Supplementary Document**. In these subgroup analyses, a positive mean difference indicates that the intervention group had higher hope scores or levels than the control group for the respective characteristic. An analysis of the intervention effects of the three videos revealed that only Video A showed significant differences across regional subgroups (p_interaction_<0.05). Although no significant heterogeneity was observed within subgroups (P_heterogeneity_>0.0083), Video A demonstrated a positive trend in boosting hope among participants from the Southern and Southwestern regions compared with the control group.

## DISCUSSION

In this nationwide, single-blind, parallel-group, randomised controlled trial, we measured the effects of three different approaches to scalable, short, animated entertainment-education videos on hope scores in a representative sample of Chinese adults. In the overall analysis, no statistically significant differences in mean hope scores were found between any of the intervention groups and the control group. However, effect sizes (Cohen's d) indicated that the intervention effects were small across all comparisons (ranging from −0.03 to 0.05), suggesting that while the animated videos may have a limited impact on hope scores at the population level, modest intervention effects cannot be ruled out. Given the strict multiple comparison correction (*P* < 0.0083) and the use of 99.17% CIs, it is possible that the study lacked sufficient statistical power to detect small yet potentially meaningful effects in certain subgroups.

### Reasons for insignificant effects

This study did not observe an overall increase in hope levels. We believe that the broader social context during the pandemic, the short intervention duration, and the characteristics of participants recruited online may have influenced the results. First, our findings contradict those of a more recent study, in which Video C (emotion) was found to boost hope among German adults [12]. This raises interesting additional questions, beyond those related to East-West cultural differences, about the moderating effect of the timing of intervention delivery. The study presented here was conducted in 2021, at the height of the COVID-19 pandemic when hope levels, globally, may have been intractably low [43,44]. In contrast, the German study was conducted post-pandemic in 2023, when hope levels among the general public may have been comparatively more responsive to change. It is also possible that the multi-cultural, inclusive approach to character design used in Video C may have resonated particularly well with an increasingly racially and ethnically diverse German population, compared with a relatively homogenous Chinese sample [45].

Second, the SAS videos did not achieve a statistically significant overall improvement in hope levels, which may be related to the relatively low ‘dose’ of the intervention in this study, as each video lasted less than three minutes. Research suggests that hope is not merely an emotional state but a cognitive process that develops through continuous interaction and iteration between pathways thinking and agency thinking. This process is typically accumulated over time and is difficult to significantly enhance through short-term interventions. Instead, it requires systematic and sustained psychological interventions to be effectively cultivated [46,47]. However, the short duration of the intervention is a necessary design consideration for such studies, as short video interventions must be feasible for rapid dissemination on social media. Therefore, future research could explore optimisation strategies, such as incorporating booster sessions to create more sustained interventions, thereby enhancing the long-term effects and more effectively capturing the potential impact of micro-interventions [48].

Third, the lack of significant intervention effects may be related to participant characteristics. First, since the study recruited participants through an online platform, the sample may be skewed toward individuals who are more tech-savvy or familiar with digital media, with urban residents accounting for more than half of the sample. These individuals may have had more prior exposure to online short videos and educational videos, making them less sensitive to similar interventions, thereby limiting the intervention’s effectiveness [15]. At the same time, this selection effect may have excluded individuals who are less familiar with digital media but could be more influenced by such videos, such as rural residents and older adults, thereby constraining the potential impact of the intervention [17]. Additionally, most participants came from central, western, and northeastern regions, with relatively low household income. Given that these regions have lower levels of economic development and greater household financial pressures, these factors may further influence mental health conditions and the effectiveness of the intervention [49,50]. Previous studies have shown that individuals experiencing greater financial stress tend to have lower psychological resilience, and the effectiveness of short-term psychological interventions may be constrained by external socioeconomic factors [51,52]. Therefore, among populations facing significant financial stress, short video interventions may be less effective in enhancing hope, as their potential impact could be offset by deeper socioeconomic challenges.

### Subgroup analysis

In the subgroup analysis, we observed significant effects of one approach – that is, the instructional-humour approach (Video A). There is a large body of communication and marketing research suggesting that storytelling and humour can effectively engage audiences and shift mindsets [53–55]. This is because humorous narratives are highly relatable to everyday life and can help alleviate stress, making educational content more easily accepted [56]. Specifically, after viewing Video A, ‘Grandma Knows Best,’ which features a quirky grandmother as the main proponent of vaccines in her family, participants in the Southern and Southwestern regions of China showed significantly higher hope levels than participants in other regions. South China is home to Shenzhen, a city and special economic zone known for its entrepreneurial, innovative, and competition-based culture. In the media, this region has sometimes been called ‘China's Silicon Valley.’ Economic and cultural characteristics of this region may more closely resemble so-called ‘Western values,’ and therefore, may also predict a general openness to humour in the southern Chinese subgroups of our study population [57]. Research suggests that some cultural differences exist in receptivity to humour, with Western cultures potentially viewing humour more positively than traditional Chinese cultures [57]. In Video A, the main source of humour derives from the main character's use of unconventional and unexpected methods to convince her family to get vaccinated. The fact that this video boosted hope more effectively within Southern and Southwestern Chinese participants may reflect underlying cultural differences in responsiveness to humour-based communication.

Video B uses the analogy of a ‘caged bird’ to represent individuals confined to their homes during the global lockdowns of COVID-19, conveying the message that ‘vaccination is the key to regaining freedom.’ However, the extent to which an analogy resonates with an audience largely depends on their familiarity with the metaphorical imagery. If the audience lacks prior knowledge or associative understanding of the analogy, the difficulty of decoding the message increases, significantly reducing its persuasive power [58]. Moreover, during the pandemic, high stress levels and information overload made the public more inclined to accept clear and direct authoritative health information [59]. Research has shown that ambiguous or obscure health communication can lead to misunderstanding and distrust. For example, a survey on vaccine-hesitant individuals in Germany found that over one-third of respondents became more sceptical about vaccine safety due to health information being ‘difficult to understand or insufficiently comprehensive [60].’ This suggests that clear and easily interpretable messages are essential for enhancing the credibility of health communication, whereas complex analogies or metaphorical expressions may reduce message acceptability and impact.

Video C adopts an emotion-driven approach, depicting the struggles of a socially isolated protagonist during the pandemic. However, Chinese culture places greater emphasis on collectivist narratives rather than focusing on individual emotional suffering and experiences of loneliness. Research indicates that during the pandemic, Chinese audiences were more likely to engage with health messages that emphasised collective values rather than narratives centred on individual hardships [16]. Furthermore, in a collectivist cultural context, people generally tend to suppress the expression of negative emotions, which may have contributed to the video’s limited effectiveness in resonating with the audience [61].

### Limitations

This study's strengths lie in its robust design, with a randomised controlled trial ensuring high internal validity and a large, diverse sample enhancing statistical power. However, several limitations should be acknowledged. First, our sample was recruited through online platforms and may not fully represent the broader Chinese population, particularly individuals with limited digital access, such as older adults and those in rural areas. Although quota sampling was used to ensure diversity across key demographic characteristics such as age, gender, and urban-rural residence, this approach cannot entirely eliminate selection bias. Future studies could employ a combination of recruitment strategies, including offline approaches, to better capture underrepresented populations. However, given that short, animated entertainment-education videos are primarily disseminated via social media, our sample is likely well-suited for testing such interventions, as it closely approximates the actual target audience. Second, our evaluation primarily focused on the immediate impact of the intervention on hope levels, without follow-up assessments to examine the persistence of its effects. While measuring immediate responses provides valuable insights into short-term changes, the long-term sustainability of the intervention's effects remains uncertain. Future research should incorporate longitudinal follow-ups to assess the durability of these effects. Finally, the use of self-reported measures may introduce social desirability bias or recall errors, potentially influencing participants' responses. While such limitations are common in survey-based studies, future research could integrate objective behavioural measures to provide a more accurate assessment of intervention effectiveness.

### Recommendations for improvement

Future efforts should focus on further improving and expanding the use of short animated video interventions to enhance their practical applicability and scalability. First, narrative techniques can be optimised by incorporating more universally relatable social roles or allegorical stories to improve cross-cultural applicability, making humour, emotional expression, and analogies more aligned with Chinese cultural contexts. Second, broader testing should be conducted to reduce potential biases introduced by online recruitment. Future studies could utilise more nationally representative survey data or offline experiments to validate intervention effectiveness. Additionally, dissemination strategies should be optimised by deploying videos across various platforms (*e.g*. WeChat, Douyin) to assess audience reception across different demographic groups. In terms of intervention design, more flexible strategies should be explored, such as testing different intervention durations to determine the optimal format, comparing the effects of multiple exposures *vs*. single viewings, and using randomised follow-up assessments to evaluate the persistence of intervention effects, allowing for a more comprehensive understanding of its impact mechanisms. Finally, to accommodate diverse cultural contexts and health concerns, it is essential to conduct thorough research on the languages, customs, and health challenges of different countries and regions. Video content and narrative styles should be adapted accordingly to align with local sociocultural contexts, thereby improving the acceptability and effectiveness of the intervention.

## CONCLUSIONS

Although the intervention effects of single-exposure, short-duration, language-free SAS videos were limited in this study, the increasing global socio-political instability and rising levels of loneliness underscore the growing importance of exploring scalable and accessible interventions to enhance hope in the post-pandemic era. Future research should continue to refine narrative techniques, test interventions across more diverse populations, and design more flexible intervention formats. Additionally, further exploration is needed to assess the long-term effects of such interventions, fully leverage their potential for cultural adaptation in health communication strategies, and enhance their applicability in a global context.

## Additional material


Online Supplementary Document
