# Supplementary material for: Effects of scalable, wordless, short, animated storytelling videos on hope in China: a nationwide, single-blind, parallel-group, randomised controlled trial
Source: J Glob Health. 2025 Jun 20;15:04140. doi: 10.7189/jogh.15.04140 (PMC12180100; doi:10.7189/jogh.15.04140)
Supplement: Online Supplementary Document [file jogh-15-04140-s001.pdf]

Supplement to: Chen W, Adam M, Geldsetzer P, Jiao L, Gates J, Zhao J, Bärnighausen T, Chen S, Wang C. Effects of scalable, wordless, short, animated storytelling videos on hope in China: a nationwide, single-blind, parallel-group, randomised controlled trial. J Glob Health. 2025;15:04140.

Content

Text S1: Quota sampling process 2

Figure S1. Study flowchart 3

Figure S2: Nationwide reach of participants in China 4

Figure S3: Effects of animated videos on proportion of high hope level 5

Figure S4: Effects of animated video A on hope score across subgroups 6

Figure S5: Effects of animated video B on hope score across subgroups 7

Figure S6: Effects of animated video C on hope score across subgroups 8

Figure S7: Effects of animated video A on proportion of high hope level across subgroups 9

Figure S8: Effects of animated video B on proportion of high hope level across subgroups 10

Figure S9: Effects of animated video C on proportion of high hope level across subgroups 11

Text S1: Quota sampling process

1. For the recruitment of 12,000 participants, each of the 31 provinces, autonomous regions, and municipalities in China was initially assigned an average quota of 360 participants.
2. Regions with populations over 40 million were assigned a larger quota of 400 participants.
3. Using the 2019 population estimates from the National Bureau of Statistics of China, quotas were determined by urban/rural, gender, and age categories. For Beijing, Shanghai, and Tianjin, rural male and female quotas were less than 50, prompting the following adjustments:

- Step 1: Raise the rural male and female quotas in Beijing, Shanghai, and Tianjin to 50 participants each.
- Step 2: To account for the increase in rural quotas, the participant quotas for Beijing and Shanghai were raised from 400 to 480, while Tianjin's quota was adjusted from 360 to 400, resulting in a total sample size of 12,000.
- Step 3: Calculate the urban quotas for Beijing, Shanghai, and Tianjin by deducting their respective rural quotas from the total, and subsequently distribute these urban quotas by gender according to the specified ratio.

Figure S1. Study flowchart

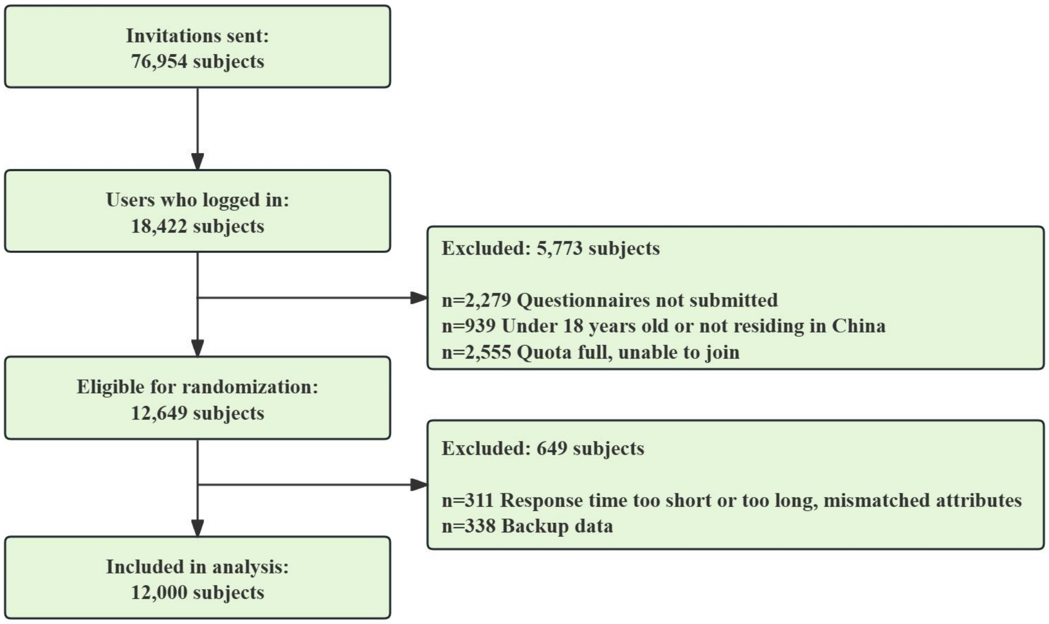

To ensure data quality, backup data were randomly chosen beyond the required sample size to replace any entries that exhibited quality issues.

Figure S2: Nationwide reach of participants in China

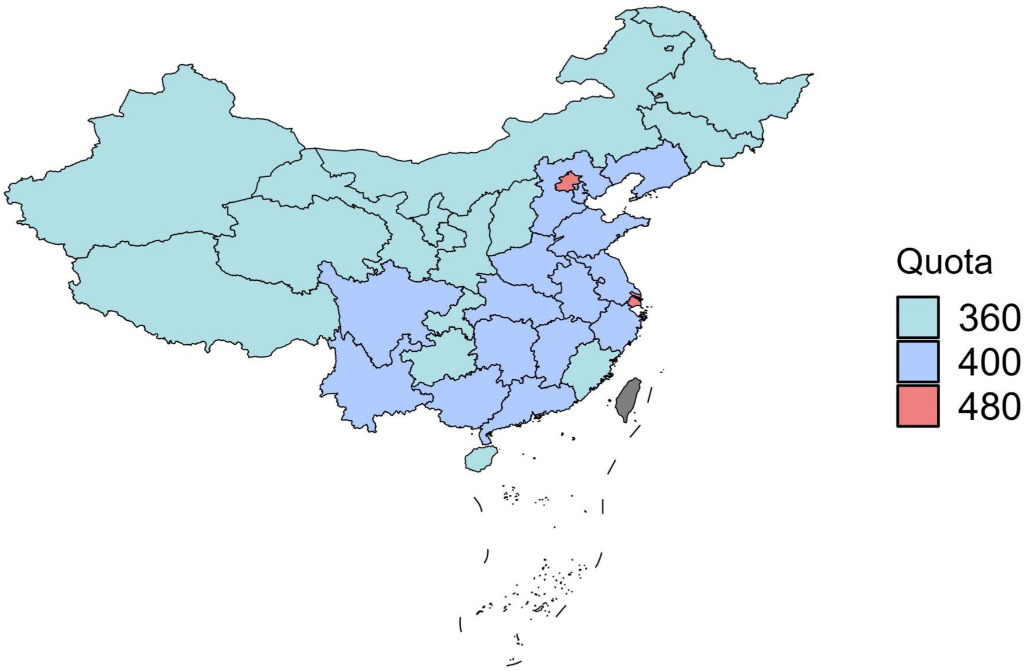

A participant quota of 360 was assigned to each of the following provinces: Shanxi, Inner Mongolia, Jilin, Heilongjiang, Fujian, Hainan, Chongqing, Guizhou, Tibet, Shaanxi, Gansu, Qinghai, Ningxia, and Xinjiang. For provinces with populations exceeding 40 million, such as Hebei, Liaoning, Jiangsu, Zhejiang, Anhui, Jiangxi, Shandong, Henan, Hubei, Hunan, Guangdong, Guangxi, Sichuan, and Yunnan, the quota was increased to 400. Meanwhile, due to their high urban population densities, the quotas for Beijing and Shanghai were set at 480, and Tianjin's was adjusted to 400.

Figure S3: Effects of animated videos on proportion of high hope level

Group	Intervention group, n/N (%)	Control group, n/N (%)	Difference (99.17% CI)	Relative risk (99.17% CI)	P value
Video A vs Control group	1774 / 3000 (59.13%)	1740 / 3000 (58.00%)	1.13 (-2.22 to 4.49)	1.02 (0.96 to 1.08)	0.37
Video B vs Control group	1763 / 3000 (58.77%)	1740 / 3000 (58.00%)	0.77 (-2.59 to 4.13)	1.01 (0.96 to 1.07)	0.55
Video C vs Control group	1754 / 3000 (58.47%)	1740 / 3000 (58.00%)	0.47 (-2.89 to 3.83)	1.01 (0.95 to 1.07)	0.71
Video A vs Video B	1774 / 3000 (59.13%)	1763 / 3000 (58.77%)	0.37 (-2.99 to 3.72)	1.01 (0.95 to 1.07)	0.77
Video A vs Video C	1774 / 3000 (59.13%)	1754 / 3000 (58.47%)	0.67 (-2.69 to 4.02)	1.01 (0.96 to 1.07)	0.60
Video B vs Video C	1763 / 3000 (58.77%)	1754 / 3000 (58.47%)	0.30 (-3.06 to 3.66)	1.01 (0.95 to 1.06)	0.81

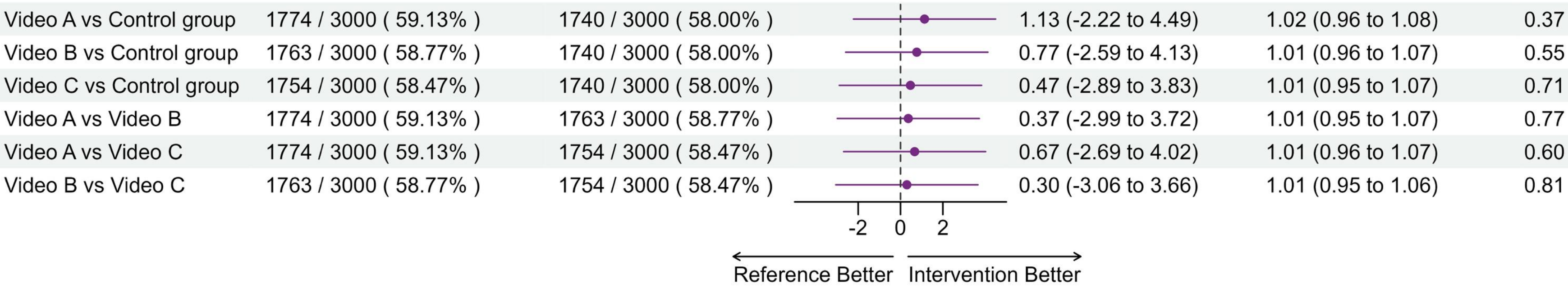

We made six comparisons, adjusting for multiplicity with an α-level of 0.0083 for each comparison. Nominal 99.17% CIs are reported to maintain an overall α-level of 0.05.

Figure S4: Effects of animated video A on hope score across subgroups

Subgroup	Video A, n (Mean, SD)	Control group, n (Mean, SD)	Difference (99.17% CI)	Cohen's d (99.17% CI)	P heterogeneity	P interaction
Age group						0.26
18-29 years	578 (45.15 , 9.30)	585 (44.20 , 9.07)	0.95 (-0.48 to 2.37)	0.10 (-0.05 to 0.26)	0.08	
30-39 years	578 (48.61 , 8.36)	575 (48.54 , 8.46)	0.07 (-1.23 to 1.38)	0.01 (-0.15 to 0.16)	0.88	
40-49 years	605 (44.74 , 9.54)	593 (45.38 , 8.67)	-0.63 (-2.02 to 0.76)	-0.07 (-0.22 to 0.08)	0.23	
50-59 years	561 (45.95 , 8.64)	568 (46.22 , 8.58)	-0.27 (-1.62 to 1.08)	-0.03 (-0.19 to 0.13)	0.60	
≥60 years	678 (48.50 , 8.17)	679 (48.45 , 8.30)	0.05 (-1.13 to 1.23)	0.01 (-0.14 to 0.15)	0.90	
Gender						0.45
Male	1521 (46.85 , 9.01)	1504 (46.65 , 8.75)	0.20 (-0.65 to 1.06)	0.02 (-0.07 to 0.12)	0.53	
Female	1479 (46.43 , 8.89)	1496 (46.57 , 8.81)	-0.14 (-1.00 to 0.71)	-0.02 (-0.11 to 0.08)	0.66	
Residence						0.15
Rural	1211 (45.58 , 9.47)	1163 (45.93 , 8.98)	-0.35 (-1.35 to 0.65)	-0.04 (-0.15 to 0.07)	0.36	
Urban	1789 (47.36 , 8.52)	1837 (47.04 , 8.62)	0.32 (-0.43 to 1.07)	0.04 (-0.05 to 0.13)	0.26	
Education						0.59
Junior high school or below	903 (47.37 , 8.82)	866 (47.68 , 8.47)	-0.30 (-1.39 to 0.78)	-0.03 (-0.16 to 0.09)	0.46	
High school or technical secondary school	965 (45.14 , 9.56)	994 (44.85 , 9.11)	0.29 (-0.82 to 1.41)	0.03 (-0.09 to 0.15)	0.48	
College diploma or above	1132 (47.34 , 8.36)	1140 (47.34 , 8.46)	0.00 (-0.93 to 0.93)	0.00 (-0.11 to 0.11)	1.00	
Total household income						0.72
Below 90,000 CNY	1118 (44.90 , 9.76)	1102 (45.00 , 9.42)	-0.10 (-1.17 to 0.97)	-0.01 (-0.12 to 0.10)	0.81	
90,000 to 180,000 CNY	1306 (46.80 , 8.43)	1296 (46.52 , 8.32)	0.28 (-0.59 to 1.14)	0.03 (-0.07 to 0.14)	0.40	
Above 180,000 CNY	576 (49.66 , 7.54)	602 (49.74 , 7.60)	-0.07 (-1.24 to 1.09)	-0.01 (-0.16 to 0.14)	0.87	
Region						0.27
Northeast China	312 (46.68 , 8.82)	272 (47.23 , 8.40)	-0.55 (-2.43 to 1.33)	-0.06 (-0.28 to 0.16)	0.44	
East China	679 (46.48 , 8.96)	717 (46.44 , 8.73)	0.04 (-1.21 to 1.29)	0.00 (-0.14 to 0.15)	0.94	
North China	507 (46.55 , 8.97)	504 (46.91 , 8.99)	-0.36 (-1.86 to 1.13)	-0.04 (-0.21 to 0.13)	0.52	
Central China	299 (45.63 , 8.71)	313 (45.79 , 8.43)	-0.16 (-1.99 to 1.67)	-0.02 (-0.23 to 0.19)	0.82	
South China	309 (47.37 , 8.68)	282 (46.40 , 8.66)	0.97 (-0.92 to 2.85)	0.11 (-0.11 to 0.33)	0.18	
Southwest China	463 (47.24 , 8.99)	453 (46.19 , 8.91)	1.05 (-0.51 to 2.61)	0.12 (-0.06 to 0.29)	0.08	
Northwest China	431 (46.51 , 9.31)	459 (47.26 , 8.95)	-0.75 (-2.37 to 0.87)	-0.08 (-0.26 to 0.10)	0.22	
Economic belt						0.74
East China	1022 (46.68 , 9.20)	1024 (46.68 , 9.01)	-0.00 (-1.07 to 1.06)	-0.00 (-0.12 to 0.12)	0.99	
Central China	583 (46.02 , 8.49)	586 (46.16 , 8.36)	-0.14 (-1.44 to 1.16)	-0.02 (-0.17 to 0.14)	0.78	
Northeast China	312 (46.68 , 8.82)	272 (47.23 , 8.40)	-0.55 (-2.43 to 1.33)	-0.06 (-0.28 to 0.16)	0.44	
West China	1083 (46.93 , 8.99)	1118 (46.63 , 8.85)	0.30 (-0.70 to 1.31)	0.03 (-0.08 to 0.15)	0.43	
**Overall**	**3000 (46.64 , 8.96)**	**3000 (46.61 , 8.77)**	**0.03 (-0.57 to 0.64)**	**0.00 (-0.06 to 0.07)**	**0.89**	

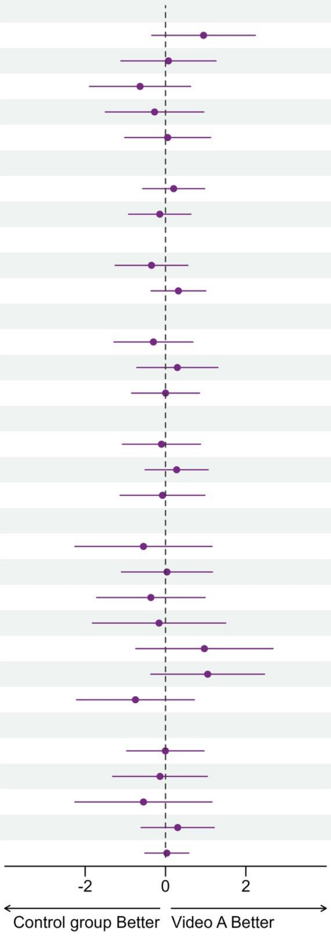

Interaction p-values below 0.05 were deemed statistically significant.

Figure S5: Effects of animated video B on hope score across subgroups

Subgroup	Video B, n (Mean, SD)	Control group, n (Mean, SD)	Difference (99.17% CI)	Cohen's d (99.17% CI)	P heterogeneity	P interaction
Age group						0.30
18-29 years	558 (45.24 , 9.11)	585 (44.20 , 9.07)	1.04 (-0.38 to 2.46)	0.11 (-0.04 to 0.27)	0.05	
30-39 years	621 (48.99 , 8.11)	575 (48.54 , 8.46)	0.45 (-0.82 to 1.72)	0.05 (-0.10 to 0.21)	0.35	
40-49 years	589 (45.02 , 9.10)	593 (45.38 , 8.67)	-0.36 (-1.72 to 1.01)	-0.04 (-0.19 to 0.11)	0.49	
50-59 years	590 (46.60 , 8.74)	568 (46.22 , 8.58)	0.37 (-0.97 to 1.72)	0.04 (-0.11 to 0.20)	0.46	
≥60 years	642 (48.27 , 8.34)	679 (48.45 , 8.30)	-0.18 (-1.38 to 1.03)	-0.02 (-0.17 to 0.12)	0.70	
Gender						0.55
Male	1578 (47.05 , 8.78)	1504 (46.65 , 8.75)	0.41 (-0.43 to 1.24)	0.05 (-0.05 to 0.14)	0.20	
Female	1422 (46.70 , 8.84)	1496 (46.57 , 8.81)	0.14 (-0.73 to 1.00)	0.02 (-0.08 to 0.11)	0.68	
Residence						0.58
Rural	1210 (46.08 , 9.22)	1163 (45.93 , 8.98)	0.14 (-0.84 to 1.13)	0.02 (-0.09 to 0.12)	0.70	
Urban	1790 (47.44 , 8.48)	1837 (47.04 , 8.62)	0.40 (-0.35 to 1.15)	0.05 (-0.04 to 0.13)	0.16	
Education						0.84
Junior high school or below	891 (47.70 , 8.60)	866 (47.68 , 8.47)	0.02 (-1.05 to 1.10)	0.00 (-0.12 to 0.13)	0.95	
High school or technical secondary school	931 (45.11 , 9.34)	994 (44.85 , 9.11)	0.27 (-0.84 to 1.38)	0.03 (-0.09 to 0.15)	0.53	
College diploma or above	1178 (47.68 , 8.32)	1140 (47.34 , 8.46)	0.34 (-0.58 to 1.26)	0.04 (-0.07 to 0.15)	0.33	
Total household income						0.43
Below 90,000 CNY	1096 (44.93 , 9.54)	1102 (45.00 , 9.42)	-0.07 (-1.14 to 1.00)	-0.01 (-0.12 to 0.11)	0.86	
90,000 to 180,000 CNY	1278 (47.09 , 8.27)	1296 (46.52 , 8.32)	0.57 (-0.29 to 1.43)	0.07 (-0.04 to 0.17)	0.08	
Above 180,000 CNY	626 (49.90 , 7.57)	602 (49.74 , 7.60)	0.16 (-0.98 to 1.31)	0.02 (-0.13 to 0.17)	0.70	
Region						0.86
Northeast China	270 (46.63 , 8.88)	272 (47.23 , 8.40)	-0.59 (-2.56 to 1.37)	-0.07 (-0.30 to 0.16)	0.42	
East China	737 (46.53 , 8.88)	717 (46.44 , 8.73)	0.08 (-1.14 to 1.30)	0.01 (-0.13 to 0.15)	0.86	
North China	478 (47.17 , 9.03)	504 (46.91 , 8.99)	0.26 (-1.26 to 1.78)	0.03 (-0.14 to 0.20)	0.65	
Central China	299 (46.14 , 8.76)	313 (45.79 , 8.43)	0.36 (-1.48 to 2.19)	0.04 (-0.17 to 0.26)	0.61	
South China	280 (46.73 , 8.49)	282 (46.40 , 8.66)	0.32 (-1.59 to 2.23)	0.04 (-0.19 to 0.26)	0.66	
Southwest China	488 (46.96 , 8.87)	453 (46.19 , 8.91)	0.77 (-0.76 to 2.30)	0.09 (-0.09 to 0.26)	0.19	
Northwest China	448 (47.85 , 8.54)	459 (47.26 , 8.95)	0.59 (-0.94 to 2.13)	0.07 (-0.11 to 0.24)	0.31	
Economic belt						0.33
East China	1019 (46.76 , 8.99)	1024 (46.68 , 9.01)	0.08 (-0.97 to 1.13)	0.01 (-0.11 to 0.13)	0.84	
Central China	595 (46.27 , 8.70)	586 (46.16 , 8.36)	0.11 (-1.20 to 1.42)	0.01 (-0.14 to 0.17)	0.83	
Northeast China	270 (46.63 , 8.88)	272 (47.23 , 8.40)	-0.59 (-2.56 to 1.37)	-0.07 (-0.30 to 0.16)	0.42	
West China	1116 (47.40 , 8.68)	1118 (46.63 , 8.85)	0.77 (-0.21 to 1.75)	0.09 (-0.02 to 0.20)	0.04	
**Overall**	**3000 (46.89 , 8.81)**	**3000 (46.61 , 8.77)**	**0.28 (-0.32 to 0.88)**	**0.03 (-0.04 to 0.10)**	**0.22**	

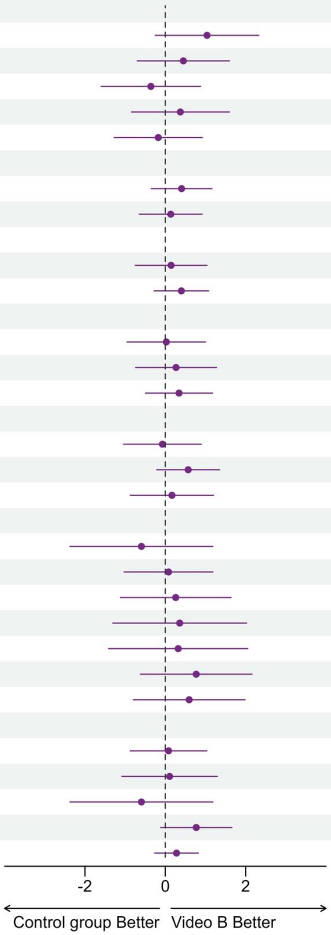

Interaction p-values below 0.05 were deemed statistically significant.

Figure S6: Effects of animated video C on hope score across subgroups

Subgroup	Video C, n (Mean, SD)	Control group, n (Mean, SD)	Difference (99.17% CI)	Cohen's d (99.17% CI)	P heterogeneity	P interaction
Age group						0.09
18-29 years	553 (45.01 , 9.32)	585 (44.20 , 9.07)	0.81 (-0.63 to 2.25)	0.09 (-0.07 to 0.24)	0.14	
30-39 years	581 (48.52 , 7.93)	575 (48.54 , 8.46)	-0.02 (-1.29 to 1.26)	-0.00 (-0.16 to 0.15)	0.97	
40-49 years	582 (44.36 , 9.15)	593 (45.38 , 8.67)	-1.02 (-2.39 to 0.35)	-0.11 (-0.27 to 0.04)	0.05	
50-59 years	571 (46.20 , 8.89)	568 (46.22 , 8.58)	-0.02 (-1.39 to 1.34)	-0.00 (-0.16 to 0.15)	0.96	
≥60 years	713 (47.70 , 8.40)	679 (48.45 , 8.30)	-0.75 (-1.93 to 0.43)	-0.09 (-0.23 to 0.05)	0.09	
Gender						0.23
Male	1524 (46.74 , 8.56)	1504 (46.65 , 8.75)	0.09 (-0.74 to 0.92)	0.01 (-0.09 to 0.11)	0.78	
Female	1476 (46.11 , 9.17)	1496 (46.57 , 8.81)	-0.46 (-1.33 to 0.41)	-0.05 (-0.15 to 0.05)	0.16	
Residence						0.27
Rural	1160 (45.43 , 9.27)	1163 (45.93 , 8.98)	-0.50 (-1.50 to 0.50)	-0.05 (-0.16 to 0.06)	0.19	
Urban	1840 (47.05 , 8.55)	1837 (47.04 , 8.62)	0.02 (-0.73 to 0.77)	0.00 (-0.08 to 0.09)	0.95	
Education						0.12
Junior high school or below	940 (46.78 , 9.09)	866 (47.68 , 8.47)	-0.90 (-1.99 to 0.19)	-0.10 (-0.23 to 0.02)	0.03	
High school or technical secondary school	958 (44.83 , 9.36)	994 (44.85 , 9.11)	-0.02 (-1.12 to 1.09)	-0.00 (-0.12 to 0.12)	0.96	
College diploma or above	1102 (47.52 , 8.00)	1140 (47.34 , 8.46)	0.19 (-0.73 to 1.11)	0.02 (-0.09 to 0.13)	0.59	
Total household income						0.59
Below 90,000 CNY	1095 (44.53 , 9.85)	1102 (45.00 , 9.42)	-0.47 (-1.56 to 0.61)	-0.05 (-0.16 to 0.06)	0.25	
90,000 to 180,000 CNY	1292 (46.57 , 8.15)	1296 (46.52 , 8.32)	0.04 (-0.81 to 0.90)	0.01 (-0.10 to 0.11)	0.90	
Above 180,000 CNY	613 (49.54 , 7.46)	602 (49.74 , 7.60)	-0.20 (-1.35 to 0.94)	-0.03 (-0.18 to 0.12)	0.64	
Region						0.76
Northeast China	266 (46.87 , 8.46)	272 (47.23 , 8.40)	-0.36 (-2.28 to 1.56)	-0.04 (-0.27 to 0.19)	0.62	
East China	707 (45.69 , 9.16)	717 (46.44 , 8.73)	-0.75 (-2.00 to 0.50)	-0.08 (-0.22 to 0.06)	0.11	
North China	511 (46.56 , 9.24)	504 (46.91 , 8.99)	-0.36 (-1.87 to 1.15)	-0.04 (-0.20 to 0.13)	0.53	
Central China	289 (46.26 , 8.34)	313 (45.79 , 8.43)	0.47 (-1.33 to 2.27)	0.06 (-0.16 to 0.27)	0.49	
South China	289 (46.16 , 9.15)	282 (46.40 , 8.66)	-0.25 (-2.21 to 1.72)	-0.03 (-0.25 to 0.19)	0.74	
Southwest China	476 (46.37 , 8.75)	453 (46.19 , 8.91)	0.17 (-1.36 to 1.70)	0.02 (-0.15 to 0.19)	0.76	
Northwest China	462 (47.49 , 8.40)	459 (47.26 , 8.95)	0.23 (-1.28 to 1.74)	0.03 (-0.15 to 0.20)	0.68	
Economic belt						0.88
East China	1015 (46.46 , 9.30)	1024 (46.68 , 9.01)	-0.23 (-1.30 to 0.85)	-0.02 (-0.14 to 0.09)	0.58	
Central China	596 (45.73 , 8.61)	586 (46.16 , 8.36)	-0.43 (-1.73 to 0.87)	-0.05 (-0.20 to 0.10)	0.38	
Northeast China	266 (46.87 , 8.46)	272 (47.23 , 8.40)	-0.36 (-2.28 to 1.56)	-0.04 (-0.27 to 0.19)	0.62	
West China	1123 (46.67 , 8.69)	1118 (46.63 , 8.85)	0.04 (-0.93 to 1.02)	0.00 (-0.11 to 0.12)	0.91	
**Overall**	**3000 (46.43 , 8.87)**	**3000 (46.61 , 8.77)**	**-0.18 (-0.78 to 0.42)**	**-0.02 (-0.09 to 0.05)**	**0.43**	

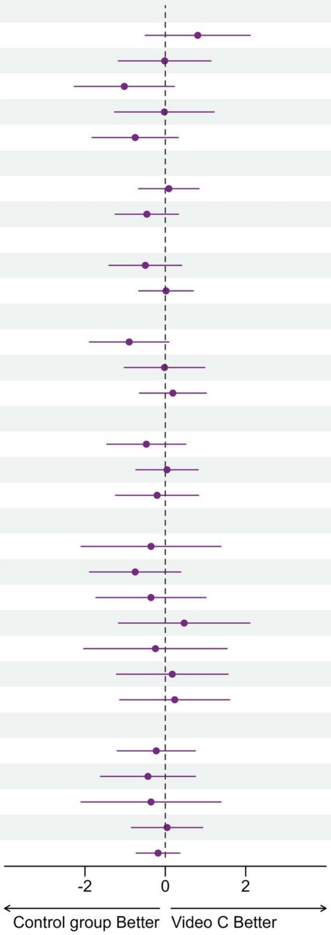

Interaction p-values below 0.05 were deemed statistically significant.

Figure S7: Effects of animated video A on proportion of high hope level across subgroups

Subgroup	Video A, n/N (%)	Control group, n/N (%)	Difference (99.17% CI)	Relative risk (99.17% CI)	P heterogeneity	P interaction
Age group						0.07
18-29 years	303 / 578 (52.42%)	265 / 585 (45.30%)	7.12 (-0.60 to 14.84)	1.16 (0.99 to 1.36)	0.02	
30-39 years	397 / 578 (68.69%)	380 / 575 (66.09%)	2.60 (-4.69 to 9.88)	1.04 (0.93 to 1.16)	0.35	
40-49 years	303 / 605 (50.08%)	314 / 593 (52.95%)	-2.87 (-10.49 to 4.75)	0.95 (0.82 to 1.10)	0.32	
50-59 years	297 / 561 (52.94%)	319 / 568 (56.16%)	-3.22 (-11.04 to 4.60)	0.94 (0.82 to 1.09)	0.28	
≥60 years	474 / 678 (69.91%)	462 / 679 (68.04%)	1.87 (-4.76 to 8.50)	1.03 (0.93 to 1.13)	0.46	
Gender						0.79
Male	905 / 1521 (59.50%)	883 / 1504 (58.71%)	0.79 (-3.93 to 5.51)	1.01 (0.94 to 1.10)	0.66	
Female	869 / 1479 (58.76%)	857 / 1496 (57.29%)	1.47 (-3.31 to 6.25)	1.03 (0.94 to 1.11)	0.42	
Residence						0.70
Rural	661 / 1211 (54.58%)	627 / 1163 (53.91%)	0.67 (-4.73 to 6.07)	1.01 (0.92 to 1.12)	0.74	
Urban	1113 / 1789 (62.21%)	1113 / 1837 (60.59%)	1.63 (-2.64 to 5.89)	1.03 (0.96 to 1.10)	0.31	
Education						0.62
Junior high school or below	581 / 903 (64.34%)	555 / 866 (64.09%)	0.25 (-5.77 to 6.27)	1.00 (0.91 to 1.10)	0.91	
High school or technical secondary school	496 / 965 (51.40%)	483 / 994 (48.59%)	2.81 (-3.15 to 8.77)	1.06 (0.94 to 1.19)	0.21	
College diploma or above	697 / 1132 (61.57%)	702 / 1140 (61.58%)	-0.01 (-5.39 to 5.38)	1.00 (0.92 to 1.09)	1.00	
Total household income						0.41
Below 90,000 CNY	575 / 1118 (51.43%)	557 / 1102 (50.54%)	0.89 (-4.71 to 6.49)	1.02 (0.91 to 1.14)	0.68	
90,000 to 180,000 CNY	787 / 1306 (60.26%)	743 / 1296 (57.33%)	2.93 (-2.16 to 8.02)	1.05 (0.96 to 1.15)	0.13	
Above 180,000 CNY	412 / 576 (71.53%)	440 / 602 (73.09%)	-1.56 (-8.45 to 5.32)	0.98 (0.89 to 1.08)	0.55	
Region						0.0219
Northeast China	187 / 312 (59.94%)	158 / 272 (58.09%)	1.85 (-8.92 to 12.62)	1.03 (0.86 to 1.24)	0.65	
East China	382 / 679 (56.26%)	411 / 717 (57.32%)	-1.06 (-8.06 to 5.94)	0.98 (0.87 to 1.11)	0.69	
North China	294 / 507 (57.99%)	300 / 504 (59.52%)	-1.54 (-9.71 to 6.64)	0.97 (0.85 to 1.12)	0.62	
Central China	165 / 299 (55.18%)	172 / 313 (54.95%)	0.23 (-10.39 to 10.85)	1.00 (0.83 to 1.22)	0.95	
South China	210 / 309 (67.96%)	163 / 282 (57.80%)	10.16 (-0.30 to 20.62)	1.18 (0.99 to 1.39)	0.01	
Southwest China	291 / 463 (62.85%)	250 / 453 (55.19%)	7.66 (-0.89 to 16.22)	1.14 (0.98 to 1.32)	0.02	
Northwest China	245 / 431 (56.84%)	286 / 459 (62.31%)	-5.46 (-14.14 to 3.21)	0.91 (0.79 to 1.06)	0.10	
Economic belt						0.69
East China	605 / 1022 (59.20%)	603 / 1024 (58.89%)	0.31 (-5.43 to 6.05)	1.01 (0.91 to 1.11)	0.89	
Central China	322 / 583 (55.23%)	330 / 586 (56.31%)	-1.08 (-8.75 to 6.59)	0.98 (0.85 to 1.13)	0.71	
Northeast China	187 / 312 (59.94%)	158 / 272 (58.09%)	1.85 (-8.92 to 12.62)	1.03 (0.86 to 1.24)	0.65	
West China	660 / 1083 (60.94%)	649 / 1118 (58.05%)	2.89 (-2.63 to 8.41)	1.05 (0.96 to 1.15)	0.17	
**Overall**	**1774 / 3000 (59.13%)**	**1740 / 3000 (58.00%)**	**1.13 (-2.22 to 4.49)**	**1.02 (0.96 to 1.08)**	**0.37**	

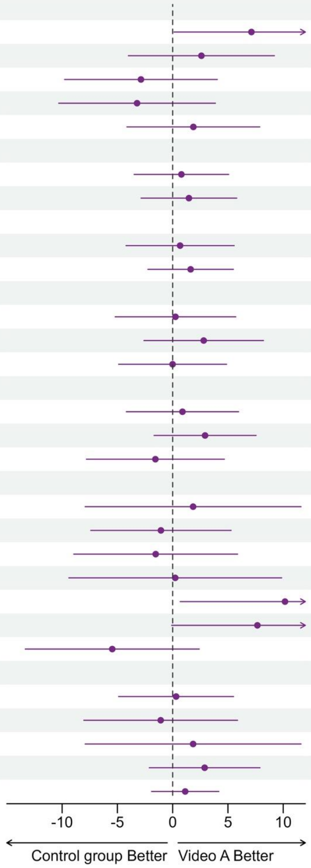

Interaction p-values below 0.05 were deemed statistically significant.

Figure S8: Effects of animated video B on proportion of high hope level across subgroups

Subgroup	Video B, n/N (%)	Control group, n/N (%)	Difference (99.17% CI)	Relative risk (99.17% CI)	P heterogeneity	P interaction
Age group						0.08
18-29 years	278 / 558 (49.82%)	265 / 585 (45.30%)	4.52 (-3.27 to 12.31)	1.10 (0.93 to 1.30)	0.13	
30-39 years	437 / 621 (70.37%)	380 / 575 (66.09%)	4.28 (-2.83 to 11.39)	1.06 (0.96 to 1.18)	0.11	
40-49 years	280 / 589 (47.54%)	314 / 593 (52.95%)	-5.41 (-13.08 to 2.25)	0.90 (0.77 to 1.05)	0.06	
50-59 years	338 / 590 (57.29%)	319 / 568 (56.16%)	1.13 (-6.56 to 8.81)	1.02 (0.89 to 1.17)	0.70	
≥60 years	430 / 642 (66.98%)	462 / 679 (68.04%)	-1.06 (-7.87 to 5.74)	0.98 (0.89 to 1.09)	0.68	
Gender						0.88
Male	935 / 1578 (59.25%)	883 / 1504 (58.71%)	0.54 (-4.14 to 5.22)	1.01 (0.93 to 1.09)	0.76	
Female	828 / 1422 (58.23%)	857 / 1496 (57.29%)	0.94 (-3.89 to 5.77)	1.02 (0.93 to 1.11)	0.61	
Residence						0.49
Rural	676 / 1210 (55.87%)	627 / 1163 (53.91%)	1.96 (-3.44 to 7.35)	1.04 (0.94 to 1.14)	0.34	
Urban	1087 / 1790 (60.73%)	1113 / 1837 (60.59%)	0.14 (-4.14 to 4.42)	1.00 (0.93 to 1.08)	0.93	
Education						0.91
Junior high school or below	568 / 891 (63.75%)	555 / 866 (64.09%)	-0.34 (-6.39 to 5.71)	0.99 (0.90 to 1.09)	0.88	
High school or technical secondary school	462 / 931 (49.62%)	483 / 994 (48.59%)	1.03 (-4.99 to 7.05)	1.02 (0.90 to 1.15)	0.65	
College diploma or above	733 / 1178 (62.22%)	702 / 1140 (61.58%)	0.65 (-4.68 to 5.97)	1.01 (0.93 to 1.10)	0.75	
Total household income						0.12
Below 90,000 CNY	524 / 1096 (47.81%)	557 / 1102 (50.54%)	-2.73 (-8.36 to 2.89)	0.95 (0.84 to 1.06)	0.20	
90,000 to 180,000 CNY	772 / 1278 (60.41%)	743 / 1296 (57.33%)	3.08 (-2.04 to 8.19)	1.05 (0.97 to 1.15)	0.11	
Above 180,000 CNY	467 / 626 (74.60%)	440 / 602 (73.09%)	1.51 (-5.11 to 8.13)	1.02 (0.93 to 1.12)	0.55	
Region						0.89
Northeast China	151 / 270 (55.93%)	158 / 272 (58.09%)	-2.16 (-13.39 to 9.06)	0.96 (0.79 to 1.17)	0.61	
East China	413 / 737 (56.04%)	411 / 717 (57.32%)	-1.28 (-8.14 to 5.58)	0.98 (0.87 to 1.10)	0.62	
North China	294 / 478 (61.51%)	300 / 504 (59.52%)	1.98 (-6.25 to 10.22)	1.03 (0.90 to 1.18)	0.53	
Central China	163 / 299 (54.52%)	172 / 313 (54.95%)	-0.44 (-11.06 to 10.19)	0.99 (0.82 to 1.20)	0.91	
South China	169 / 280 (60.36%)	163 / 282 (57.80%)	2.56 (-8.39 to 13.50)	1.04 (0.87 to 1.26)	0.54	
Southwest China	286 / 488 (58.61%)	250 / 453 (55.19%)	3.42 (-5.11 to 11.94)	1.06 (0.91 to 1.23)	0.29	
Northwest China	287 / 448 (64.06%)	286 / 459 (62.31%)	1.75 (-6.70 to 10.21)	1.03 (0.90 to 1.18)	0.58	
Economic belt						0.36
East China	599 / 1019 (58.78%)	603 / 1024 (58.89%)	-0.10 (-5.85 to 5.64)	1.00 (0.91 to 1.10)	0.96	
Central China	325 / 595 (54.62%)	330 / 586 (56.31%)	-1.69 (-9.33 to 5.94)	0.97 (0.85 to 1.11)	0.56	
Northeast China	151 / 270 (55.93%)	158 / 272 (58.09%)	-2.16 (-13.39 to 9.06)	0.96 (0.79 to 1.17)	0.61	
West China	688 / 1116 (61.65%)	649 / 1118 (58.05%)	3.60 (-1.87 to 9.07)	1.06 (0.97 to 1.16)	0.08	
**Overall**	**1763 / 3000 (58.77%)**	**1740 / 3000 (58.00%)**	**0.77 (-2.59 to 4.13)**	**1.01 (0.96 to 1.07)**	**0.55**	

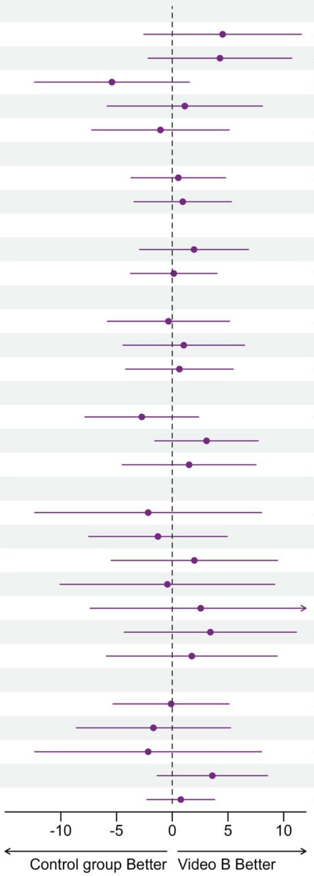

Interaction p-values below 0.05 were deemed statistically significant.

Figure S9: Effects of animated video C on proportion of high hope level across subgroups

Subgroup	Video C, n/N (%)	Control group, n/N (%)	Difference (99.17% CI)	Relative risk (99.17% CI)	P heterogeneity	P interaction
Age group						0.06
18-29 years	281 / 553 (50.81%)	265 / 585 (45.30%)	5.51 (-2.30 to 13.33)	1.12 (0.95 to 1.32)	0.06	
30-39 years	403 / 581 (69.36%)	380 / 575 (66.09%)	3.28 (-3.98 to 10.53)	1.05 (0.94 to 1.17)	0.23	
40-49 years	288 / 582 (49.48%)	314 / 593 (52.95%)	-3.47 (-11.16 to 4.23)	0.93 (0.80 to 1.09)	0.23	
50-59 years	327 / 571 (57.27%)	319 / 568 (56.16%)	1.11 (-6.64 to 8.86)	1.02 (0.89 to 1.17)	0.71	
≥60 years	455 / 713 (63.81%)	462 / 679 (68.04%)	-4.23 (-10.93 to 2.47)	0.94 (0.85 to 1.04)	0.10	
Gender						0.18
Male	927 / 1524 (60.83%)	883 / 1504 (58.71%)	2.12 (-2.59 to 6.82)	1.04 (0.96 to 1.12)	0.23	
Female	827 / 1476 (56.03%)	857 / 1496 (57.29%)	-1.26 (-6.05 to 3.54)	0.98 (0.90 to 1.06)	0.49	
Residence						0.42
Rural	616 / 1160 (53.10%)	627 / 1163 (53.91%)	-0.81 (-6.27 to 4.65)	0.98 (0.89 to 1.09)	0.70	
Urban	1138 / 1840 (61.85%)	1113 / 1837 (60.59%)	1.26 (-2.98 to 5.50)	1.02 (0.95 to 1.09)	0.43	
Education						0.05
Junior high school or below	564 / 940 (60.00%)	555 / 866 (64.09%)	-4.09 (-10.11 to 1.94)	0.94 (0.85 to 1.03)	0.07	
High school or technical secondary school	502 / 958 (52.40%)	483 / 994 (48.59%)	3.81 (-2.16 to 9.78)	1.08 (0.96 to 1.21)	0.09	
College diploma or above	688 / 1102 (62.43%)	702 / 1140 (61.58%)	0.85 (-4.56 to 6.26)	1.01 (0.93 to 1.11)	0.68	
Total household income						0.86
Below 90,000 CNY	568 / 1095 (51.87%)	557 / 1102 (50.54%)	1.33 (-4.30 to 6.96)	1.03 (0.92 to 1.15)	0.53	
90,000 to 180,000 CNY	739 / 1292 (57.20%)	743 / 1296 (57.33%)	-0.13 (-5.27 to 5.00)	1.00 (0.91 to 1.09)	0.95	
Above 180,000 CNY	447 / 613 (72.92%)	440 / 602 (73.09%)	-0.17 (-6.89 to 6.55)	1.00 (0.91 to 1.09)	0.95	
Region						0.76
Northeast China	161 / 266 (60.53%)	158 / 272 (58.09%)	2.44 (-8.74 to 13.62)	1.04 (0.86 to 1.26)	0.56	
East China	383 / 707 (54.17%)	411 / 717 (57.32%)	-3.15 (-10.10 to 3.80)	0.95 (0.83 to 1.07)	0.23	
North China	301 / 511 (58.90%)	300 / 504 (59.52%)	-0.62 (-8.76 to 7.52)	0.99 (0.86 to 1.14)	0.84	
Central China	167 / 289 (57.79%)	172 / 313 (54.95%)	2.83 (-7.84 to 13.51)	1.05 (0.87 to 1.27)	0.48	
South China	171 / 289 (59.17%)	163 / 282 (57.80%)	1.37 (-9.52 to 12.25)	1.02 (0.85 to 1.23)	0.74	
Southwest China	278 / 476 (58.40%)	250 / 453 (55.19%)	3.22 (-5.36 to 11.79)	1.06 (0.91 to 1.23)	0.32	
Northwest China	293 / 462 (63.42%)	286 / 459 (62.31%)	1.11 (-7.29 to 9.52)	1.02 (0.89 to 1.16)	0.73	
Economic belt						0.67
East China	597 / 1015 (58.82%)	603 / 1024 (58.89%)	-0.07 (-5.82 to 5.68)	1.00 (0.91 to 1.10)	0.97	
Central China	323 / 596 (54.19%)	330 / 586 (56.31%)	-2.12 (-9.75 to 5.51)	0.96 (0.84 to 1.11)	0.46	
Northeast China	161 / 266 (60.53%)	158 / 272 (58.09%)	2.44 (-8.74 to 13.62)	1.04 (0.86 to 1.26)	0.56	
West China	673 / 1123 (59.93%)	649 / 1118 (58.05%)	1.88 (-3.61 to 7.36)	1.03 (0.94 to 1.13)	0.37	
**Overall**	**1754 / 3000 (58.47%)**	**1740 / 3000 (58.00%)**	**0.47 (-2.89 to 3.83)**	**1.01 (0.95 to 1.07)**	**0.71**	

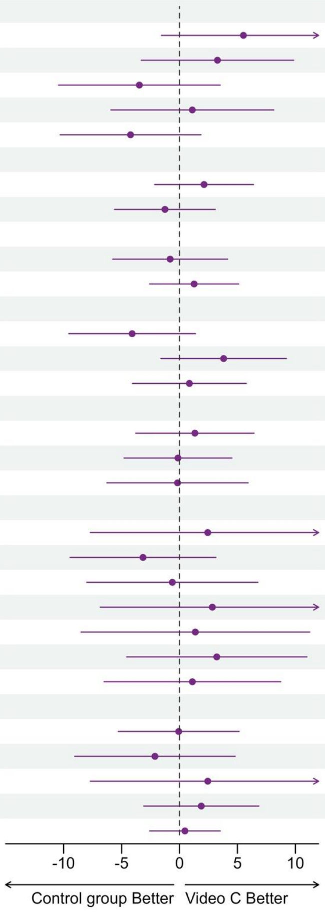

Interaction p-values below 0.05 were deemed statistically significant.
